# Supplementary material for: Efficient DNA-free genome editing of bread wheat using CRISPR/Cas9 ribonucleoprotein complexes
Source: Nat Commun. 2017 Jan 18;8:14261. doi: 10.1038/ncomms14261 (PMC5253684; doi:10.1038/ncomms14261)
Supplement: Supplementary Information — Supplementary Figures and Supplementary Tables [file ncomms14261-s1.pdf]

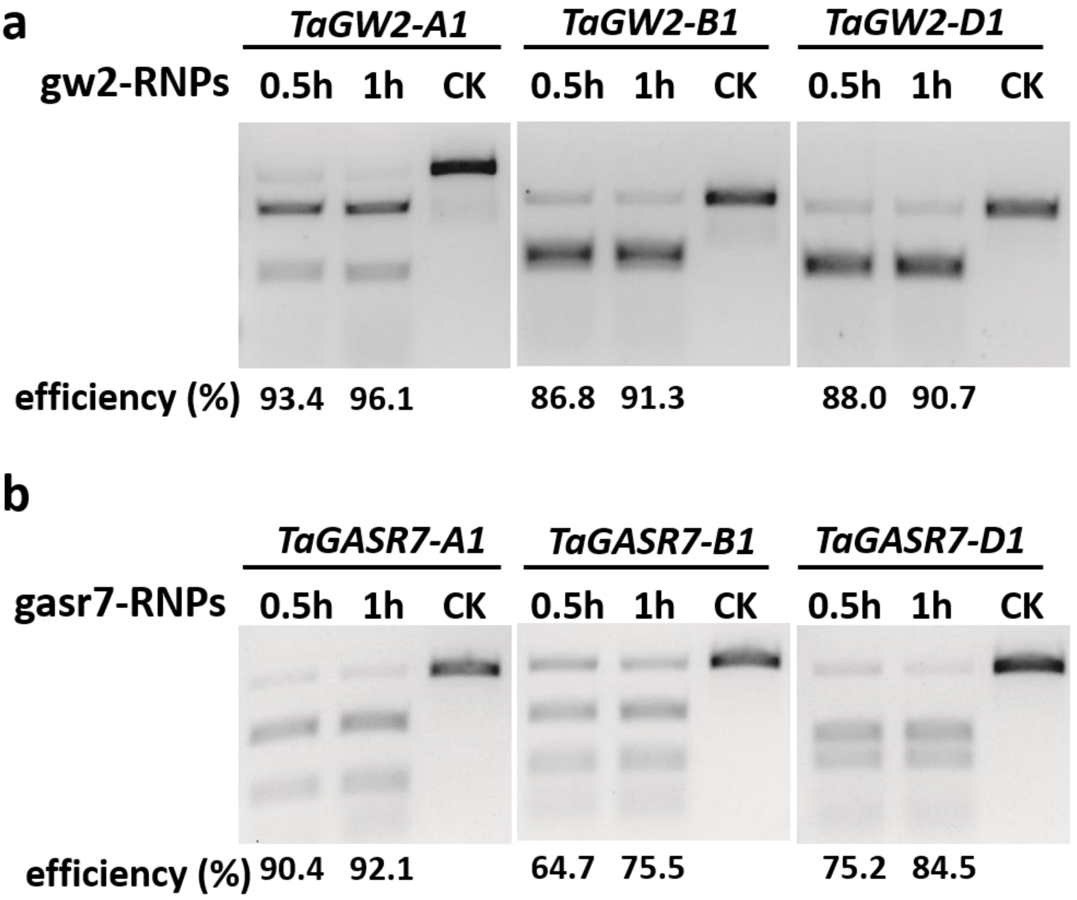

Supplementary Figure 1. *In vitro* cleavage of three homoeologs of *TaGW2* (*TaGW2-A1, -B1* and *-D1*) and *TaGASR7* (*TaGASR7-A1, -B1* and *-D1*) using gw2-RNPs or gasr7-RNPs. CK indicates the incubation without the addition of RNPs.

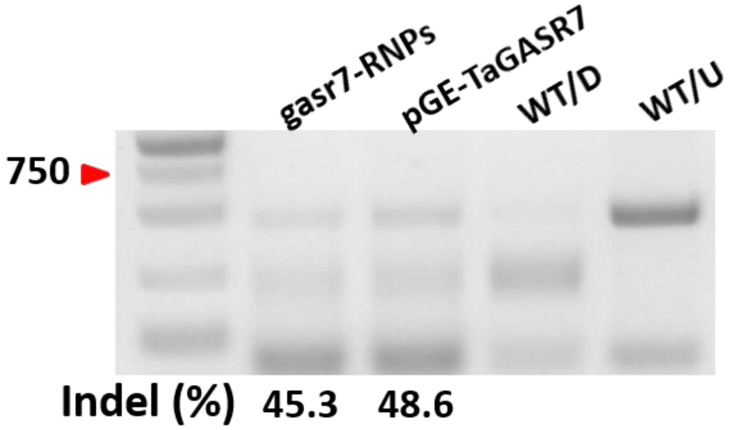

Supplementary Figure 2. Mutagenesis frequencies of *TaGASR7* (induced by gasr7-RNPs or pGE-TaGASR7) in wheat protoplasts analyzed by PCR-RE assay. WT/D and WT/U indicate wild type PCR amplicons with or without restriction enzyme digestion.

a

TaGW2-A1 (pGE-TaGW2)

```
                                      count
WT:  CCTCTAGAAATGCCCCATCCTG
M1:  CCTCTAAGAAATGCCCCATCCTG   +1  996
M2:  CCTCTATGAAATGCCCCATCCTG   +1  367
M3:  CCTCGA--AATGCCCCATCCTG    -2  330
M4:  CCTCTA----TGCCCCATCCTG    -4  284
M5:  CCTCTA-----GCCCCATCCTG    -5  276
M6:  CCTCTACGAAATGCCCCATCCTG   +1  141
M7:  CCTCT-GAAATGCCCCATCCTG    -1  129
M8:  CCTCT---AATGCCCCATCCTG    -3  120
M9:  CCTCTA-AAATGCCCCATCCTG    -1  115
M10: CCTCTAGGAAATGCCCCATCCTG   +1  105
```

TaGW2-A1 (gw2-RNPs)

```
                                      count
WT:  CCTCTAGAAATGCCCCATCCTG
M1:  CCTCTAAGAAATGCCCCATCCTG   +1  39
M2:  CCTCTATGAAATGCCCCATCCTG   +1  25
M3:  CCTCT-GAAATGCCCCATCCTG    -1  23
M4:  CCTCT--AAATGCCCCATCCTG    -2  22
M6:  CCTC--GAAATGCCCCATCCTG    -2  5
M7:  CCTCTACGAAATGCCCCATCCTG   +1  4
M8:  CCTCT----ATGCCCCATCCTG    -4  4
```

b

TaGW2-B1 (pGE-TaGW2)

```
                                      count
WT:  CCTCTAGAAATACCCCATCCTG
M1:  CCTCTAAGAAATACCCCATCCTG   +1  277
M2:  CCTCT---AATACCCCATCCTG    -3  200
M3:  CCTCTATGAAATACCCCATCCTG   +1  184
M4:  CCTCT----ATACCCCATCCTG    -4  168
M5:  CCTCT--AAATACCCCATCCTG    -2  127
M6:  CCTCTA-AAATACCCCATCCTG    -1  44
M7:  CCTCTACGAAATACCCCATCCTG   +1  38
M8:  CCTCT-GAAATACCCCATCCTG    -1  37
M9:  CCTCTA-----ACCCCATCCTG    -5  32
M10: C-----GAAATACCCCATCCTG    -5  28
```

TaGW2-B1 (gw2-RNPs)

```
                                      count
WT:  CCTCTAGAAATACCCCATCCTG
M1:  CCTCTAAGAAATACCCCATCCTG   +1     198
M2:  CCTCTATGAAATACCCCATCCTG   +1     67
M3:  CCTC--GAAATACCCCATCCTG    -2     43
M4:  CCTCT--AAATACCCCATCCTG    -2     33
M5:  CCTCTACGAAATACCCCATCCTG   +1     28
M6:  CCTCTA-AAATACCCCATCCTG    -1     21
M7:  CCTCT--AAATACCCCATCCTG    -2     20
M8:  CCTCT-GAAATACCCCATCCTG    -1     16
M9:  CCTCT---AATACCCCATCCTG    -3     12
M10: CCTCTA---TTACCCCATCCTG    -4/+1  12
```

c

TaGW2-D1 (pGE-TaGW2)

```
                                      count
WT:  CCTCTAGAAATACCCCATCCTG
M1:  CCTCTAAGAAATACCCCATCCTG   +1  906
M2:  CCTCT---AATACCCCATCCTG    -3  652
M3:  CCTCTATGAAATACCCCATCCTG   +1  515
M4:  CCTCT----ATACCCCATCCTG    -4  500
M5:  CCTCTACGAAATACCCCATCCTG   +1  212
M6:  CCTCTA-----ACCCCATCCTG    -5  172
M7:  CCTCTAGGAAATACCCCATCCTG   +1  142
M8:  CCTCTA-AAATACCCCATCCTG    -1  102
M9:  CCTCTA----TGCCCCATCCTG    -4  64
M10: CCTCTAG--ATACCCCATCCTG    -2  50
```

TaGW2-D1 (gw2-RNPs)

```
                                      count
WT:  CCTCTAGAAATACCCCATCCTG
M1:  CCTCTAAGAAATACCCCATCCTG   +1  268
M2:  CCTCTATGAAATACCCCATCCTG   +1  155
M3:  CCTCT---AATACCCCATCCTG    -3  107
M4:  CCTCT----ATACCCCATCCTG    -4  32
M5:  CCTCTACGAAATACCCCATCCTG   +1  26
M6:  CCTCT--AAATACCCCATCCTG    -2  26
M7:  CCTCTAGGAAATACCCCATCCTG   +1  23
M8:  CCT---GAAATACCCCATCCTG    -3  12
M9:  CCTC--GAAATACCCCATCCTG    -2  8
M10: CCTCT-GAAATACCCCATCCTG    -1  7
```

Supplementary Figure 3. Ten most frequently occurred mutation types generated by pGE-TaGW2 or gw2-RNPs in immature embryos revealed by deep amplicon sequencing. Hyphens denote deleted nucleotides. The nucleotides inserted are labeled green. The PAM motif (CCT) is shown in red. The number on the right sides indicates the captured reads of the mutation type.

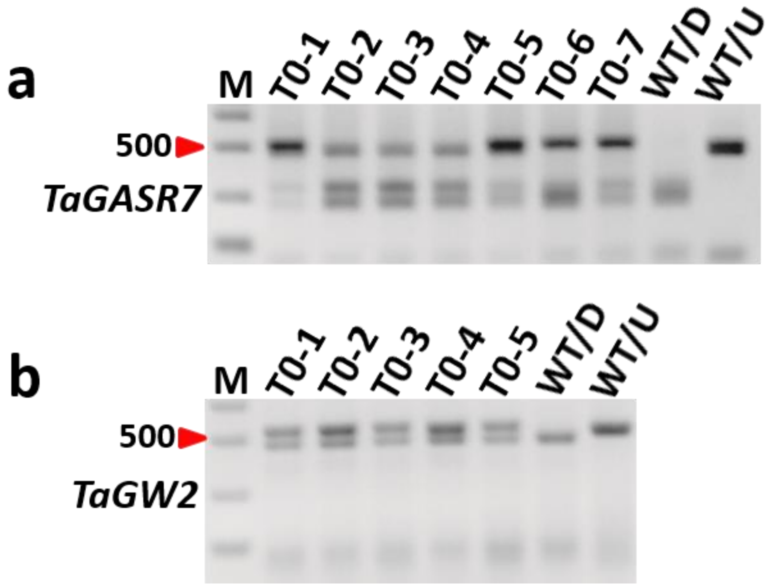

Supplementary Figure 4. PCR-RE assay analysis for gasr7-RNPs (a) and gw2-RNPs (b) induced mutants in hexaploid wheat line YZ814. Lanes T0-1 to T0-7 show the PCR products of the mutants after enzyme digestion. Lanes labeled by WT/D and WT/U are the PCR products amplified from wild type (WT) plants with and without enzyme digestion, respectively.

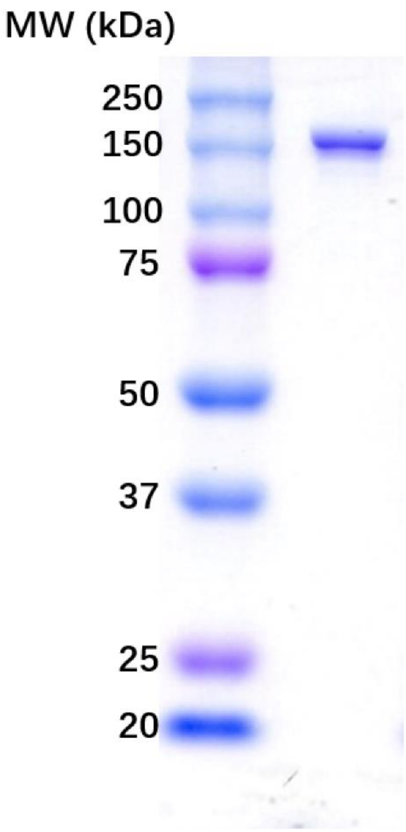

Supplementary Figure 5. SDS-PSGE image of purified Cas9 protein. 1 μl Cas9 protein was detected by SDS-PAGE electrophoresis system using 10% separating gel.

Supplementary Table 1. sgRNA target loci and sequences.

Gene name	Target site (5'-3')	Oligo-F (5'-3')	Oligo-R (5'-3')	Detection method
TaGW2	CCTCTAGAAATACCCCATCCTG	CTTGCAGGATGGGGTATTTCTAG	AAACCTAGAAATACCCCATCCTG	Deep sequencing and PCR-RE XbaI
TaGASR7	CCGCCGGGCACCTACGGCAAC	CTTGGTTGCCGTAGGTGCCCGG	AAACCCGGGCACCTACGGCAAC	Deep sequencing and PCR-RE BcnI

Supplementary Table 2. Mutagenesis frequencies of *TaGW2-A1*, *-B1*, *-D1* (induced by gw2-RNPs or pGE-TaGW2) and *TaGASR7* (induced by gasr7-RNPs or pGE-TaGASR7) in the embryos revealed by deep amplicon sequencing.

Target gene	Reagents	Total reads	Mutant reads	Mutagenesis frequency (%)
TaGW2-A1	pGE-TaGW2	425527	3245	0.76
	gw2-RNPs	390295	122	0.03
	Control	408883	10	0
TaGW2-B1	pGE-TaGW2	133533	1320	0.99
	gw2-RNPs	296538	541	0.18
	Control	220458	21	0.01
TaGW2-D1	pGE-TaGW2	433865	4333	1.00
	gw2-RNPs	323010	677	0.21
	Control	213139	19	0.01
TaGASR7	gasr7-RNPs	80240	448	0.56
	Control	17632	0	0

Supplementary Table 3. PCR primers used in this study.

Primer name	Primer sequence (5'-3')	Application
GW2-F<br>GW2-R	ATGCCAACCCTTGCGTGTGCGT<br>TCCTGCTTGTGGGAGCTTTATG	Amplifying the *TaGW2* target site
GW2-A1-F	CTGCCATTACTTTGTATTTTGGTAATA	Amplifying the *TaGW2-A1* target site and 1st PCR for deep sequencing
GW2-B1-F	GTTCAGATGGCAATCTAAAAGTT	Amplifying the *TaGW2-B1* target site and 1st PCR for deep sequencing
GW2-D1-F	GCATGTACTTTGATTGTTTGCGTGA	Amplifying the T*aGW2-D1* target site and 1st PCR for deep sequencing
GW2-A1/B1/D1-R	TCCTTCCTCTCTTACCACTTCCC	Amplifying the *TaGW2-A1, -B1 and -D1* target site and 1st PCR for deep sequencing
T7-GW2-F<br>sgRNA-PCR-R	TAATACGACTCACTATAGGCAGGATGGGGTATTTCTAG<br>GCACCGACTCGGTGCCACTT	Amplifying the T7-GW2-sgRNA
T7-GASR7-F<br>sgRNA-PCR-R	TAATACGACTCACTATAGGGTTGCCGTAGGTGCCCGG<br>GCACCGACTCGGTGCCACTT	Amplifying the T7-GASR7-sgRNA
GASR7-F<br>GASR7-R	GGAGGTGATGGGAGGTGGGGG<br>CTGGGAGGGCAATTCACATGCCA	Amplifying the *TaGASR7* target site and 1st PCR for deep sequencing
GASR7-A1/B1/D1-F	CCTTCATCCTTCAGCCATGCAT	Amplifying the *TaGASR7-A1, -B1 and -D1* target site
GASR7-A1-R	CCACTAAATGCCTATCACATACG	Amplifying the *TaGASR7-A1* target site
GASR7-B1-R	AGGGCAATTCACATGCCACTGAT	Amplifying the *TaGASR7-B1* target site
GASR7-D1-R	CCTCCATTTTTCCACATCTTAGTCC	Amplifying the *TaGASR7-D1* target site
BA1-GW2-AF<br>BA2-GW2-AR	CGATGTTGCCTTTTGAGCAACCAACG<br>TGACCATCCATGCTTGATGGCTGGTG	2nd PCR for deep sequencing of *TaGW2-A1* of pGE-TaGW2
BA3-GW2-BF<br>BA4-GW2-BR	ACAGTGTGCCTTTTGAGCAACCAACG<br>GCCAATTCCATGCTTGATGGCTGGTG	2nd PCR for deep sequencing of *TaGW2-B1* of pGE-TaGW2
BA5-GW2-DF<br>BA6-GW2-DR	CAGATCTGCCTTTTGAGCAACCAACG<br>CTTGTATCCATGCTTGCTGGCTGGTG	2nd PCR for deep sequencing of *TaGW2-D1* of pGE-TaGW2
BA13-GW2-AF<br>BA14-GW2-AR	AGTCAATGCCTTTTGAGCAACCAACG<br>AGTTCCTCCATGCTTGATGGCTGGTG	2nd PCR for deep sequencing of *TaGW2-A1* of gw2-RNPs
BA15-GW2-BF<br>BA16-GW2-BR	ATGTCATGCCTTTTGAGCAACCAACG<br>CCGTCCTCCATGCTTGATGGCTGGTG	2nd PCR for deep sequencing of *TaGW2-B1* of gw2-RNPs
BA17-GW2-DF<br>BA18-GW2-DR	GTAGAGTGCCTTTTGAGCAACCAACG<br>GTCCGCTCCATGCTTGCTGGCTGGTG	2nd PCR for deep sequencing of *TaGW2-D1* of gw2-RNPs
BA43-GW2-AF<br>BA44-GW2-AR	TACAGCTGCCTTTTGAGCAACCAACG<br>TATAATTCCATGCTTGATGGCTGGTG	2nd PCR for deep sequencing of *TaGW2-A1* of WT
BA45-GW2-BF<br>BA46-GW2-BR	TCATTCTGCCTTTTGAGCAACCAACG<br>TCCCGATCCATGCTTGATGGCTGGTG	2nd PCR for deep sequencing of *TaGW2-B1* of WT
BA47-GW2-DF<br>BA48-GW2-DR	TCGAAGTGCCTTTTGAGCAACCAACG<br>TCGGCATCCATGCTTGCTGGCTGGTG	2nd PCR for deep sequencing of *TaGW2-D1* of WT
BA41-GASR7-F<br>BA42-GASR7-R	GCGCTAGAAGGCGTGCCTGACCTACT<br>TAATCGCAAGAATCTAGGGGCACTTGG	2nd PCR for deep sequencing of *TaGASR7* of WT
BA43-GASR7-F<br>BA44-GASR7-R	TACAGCGAAGGCGTGCCTGACCTACT<br>TATAATCAAGAATCTAGGGGCACTTGG	2nd PCR for deep sequencing of *TaGASR7* of gasr7-RNPs

Supplementary Table 4. Genotypes of another 16 *tagw2* mutant plants induced by gw2-RNPs.

Plant ID	Genotype of *TaGW2-A1*	Genotype of *TaGW2-B1*	Genotype of *TaGW2-D1*
T0-13	AA	BB	Dd (-3 bp)
T0-14	AA	Bb (+1 bp)	dd (-9 bp)
T0-15	AA	Bb (+1 bp)	dd (-9 bp)
T0-16	AA	Bb (-1 bp)	Dd (+1 bp)
T0-17	AA	Bb (-1 bp)	Dd (+1 bp)
T0-18	AA	BB	Dd (-16 bp)
T0-19	AA	Bb (-6/+1 bp)	Dd (-6 bp)
T0-20	AA	Bb (-6/+1 bp)	Dd (-6 bp)
T0-21	AA	Bb (-6/+1 bp)	Dd (-6 bp)
T0-22	AA	BB	Dd (+1 bp)
T0-23	AA	BB	Dd (+1 bp)
T0-24	AA	BB	Dd (+1 bp)
T0-25	AA	BB	Dd (-3 bp)
T0-26	AA	BB	Dd (-3 bp)
T0-27	AA	BB	Dd (-6 bp)
T0-28	AA	BB	Dd (-6 bp)

Supplementary Table 5. Potential off-target sites analyzed for *TaGW2*.

Site name	Sequence	No. of mismatches	Mutagenesis frequency	Detection Method
On-target	CCTCTAGAAATACCCCATCCTG			
OT1	gAGGATGGGGTATTTaTAGTGG	2	0	Sanger Sequencing
OT2	tAaGATGGGtTATTTCTAGAGG	3	0	PCR-RE XbaI
OT3	gAGGATGGGaTtTTTCTAGAGG	3	0	PCR-RE XbaI
OT4	gAGGAgGGGGTATTTaTAGTGG	3	0	Sanger Sequencing
OT5	tAaGATGGGtTATTTCTAGAGG	3	0	Sanger Sequencing
OT6	CAGcATcGGGTATTTCTcGAGG	3	0	Sanger Sequencing
OT7	CtGGAcGGGGTATTTgTAGTGG	3	0	Sanger Sequencing
OT8	CAGGgTtGGGTcTTTCTAGTGG	3	0	Sanger Sequencing
OT9	gAGGcTGGGGTATTTaTAGTGG	3	0	Sanger Sequencing
OT10	CAcGATGGcGcATTTCTAGTGG	3	0	Sanger Sequencing
OT11	aAGaATGGGccATTTCTAGAGG	4	0	PCR-RE XbaI
OT12	aAGaATGGGtcATTTCTAGAGG	4	0	PCR-RE XbaI
OT13	CgGcATGGaGTATaTCTAGAGG	4	0	PCR-RE XbaI
OT14	CcGGAgGGaGTATgTCTAGAGG	4	0	PCR-RE XbaI
OT15	ttGGAaGGGGgATTTCTAGAGG	4	0	PCR-RE XbaI
OT16	gAGGAcaGaGgATTTCTAGAGG	5	0	PCR-RE XbaI
OT17	CttGtgtGGGTATTTCTAGTGG	5	0	Sanger Sequencing
OT18	CAtGgTaGaGTtTTTCTAGTGG	5	0	Sanger Sequencing
OT18	tAaGATtGGGTATTgCTAtAGG	5	0	Sanger Sequencing
OT20	gAGcAgGcGGTATTTaTAGTGG	5	0	Sanger Sequencing
